# Microsurgery versus embolization: different risk factors for short- and longterm outcomes of patients with ruptured aneurysms

**DOI:** 10.1590/acb370806

**Published:** 2022-11-28

**Authors:** Marcia Harumy Yoshikawa, Nícollas Nunes Rabelo, João Paulo Mota Telles, Guilherme Bitencourt Barbosa, Natália Camargo Barbato, Antônio Carlos Samaia da Silva Coelho, Leonardo Zumerkorn Pipek, Manoel Jacobsen Teixeira, Eberval Gadelha Figueiredo

**Affiliations:** 1 MS. Universidade Federal de São Paulo – School of Medicine – São Paulo (SP), Brazil.; 2 PhD. Universidade Federal de São Paulo – Department of Neurosurgery – São Paulo (SP), Brazil.; 3 MS. Universidade Federal do ABC – School of Medicine – Santo André (SP), Brazil.

**Keywords:** Intracranial Aneurysm, Subarachnoid Hemorrhage, Plaque, Atherosclerotic, Aneurysms, Follow-Up Studies, Prognosis

## Abstract

**Purpose:**

To evaluate the risk factors for poor outcomes after surgical and endovascular treatment of aneurysmal subarachnoid hemorrhage (aSAH).

**Methods:**

Patients with ≥ 18-years of age and aSAH were included, while patients who died within 12 h of admission or lost follow-up were excluded. All participants underwent standardized clinical and radiological assessment on admission and were reassessed at discharge and at 6-months follow-up using the Glasgow Outcome Scale (GOS).

**Results:**

Death at discharge was associated with female gender, anterior communication artery (ACoA) aneurysm location and presence of atherosclerotic plaque in the surgical group, and with age in the endovascular group. Both groups had clinical condition on follow-up associated with mFisher score on admission and hypertension. GOS on follow-up was also associated with presence of atherosclerotic plaque and multiple aneurysms in surgical group, and with age in endovascular group.

**Conclusions:**

Subjects treated surgically are prone to unfavorable outcomes if atherosclerotic plaques and multiple aneurysms are present. In patients with endovascular treatment, age was the main predictor of clinical outcome.

## Introduction

Subarachnoid hemorrhage (SAH) is a subset of stroke that occurs in relatively young patients and that have poor prognosis in most cases: about 50% dies in the first month and 40% of those who survive remains functionally dependent^1^,^2^. SAH secondary to rupture of intracranial aneurysms, the aneurysmal subarachnoid hemorrhage (aSAH), accounts for 80% of nontraumatic SAH cases^1^,^2^ and its management may be endovascular or surgical.

For the last two decades, the treatment of SAH secondary to aneurysm rupture has changed due to the increasingly use of endovascular coiling, especially after the results of the International Subarachnoid Aneurysm Trial (ISAT)^3^. The ISAT demonstrated that endovascular treatment of patients with ruptured intracranial aneurysms and good clinical condition on admission leads to better short and long-term outcomes than surgical clipping^3^,^4^.

Following studies have investigated the subpopulations in which coiling or clipping would be more beneficial. According to Pierot *et al*.^5^, risk factors for procedural complications during endovascular coiling include aneurysm’s size, neck size and location, age, smoking and hypertension. Nguyen *et al*.^6^ studied the use of endovascular treatment in very small aneurysms and found that this procedure is five times more likely to results in procedure-related rupture when performed in aneurysms < 3 mm than in larger lesions.

Even though the risk factors for poor outcomes after the surgical treatment of ruptured aneurysms are well known (which include severity of the initial event, rebleeding, perioperative medical management, and the timing and technical success of aneurysm treatment), evidence regarding the risk factors for poor outcomes after endovascular coiling is still lacking. Therefore, considering the importance of comparing the different risk factor to better indicate the treatment, the aim of this study is to assess risk factors for poor outcomes after surgical and endovascular treatment of ruptured intracranial aneurysms.

## Methods

This prospective cohort study was performed in Central Institute of Clinical Hospital from School of Medicine, University of São Paulo (HC-FMUSP), and was approved by the Ethics and Research Committee of HC-FMUSP. All methods were performed in accordance with the relevant guidelines and regulations. Each patient was fully informed about the study and provided informed consent as approved by the local Institutional Review Board.

### Patient selection

This study enrolled patients from HC-FMUSP Division of Neurological Surgery between January 2018 and November 2019. Patients were eligible for enrollment if they were 18 years of age or older and admitted in the emergence department of the Central Institute of HC-FMUSP with diagnosis of ruptured aneurysm. Patients who died within the first 12 h after admission or lost follow-up after hospital discharge were excluded.

### Data collection

Participants’ characteristics assessed included age, gender, hypertension, diabetes mellitus, smoking, alcohol abuse and previous SAH. The clinical condition at admission was assessed through the Hunt–Hess scale (HH). The radiological assessment included the modified Fisher classification (mFisher) on admission, multiple aneurysms, aneurysm’s location and size, and presence of atherosclerotic plaque in intracranial arteries. Time from ictus until intervention, clinical and surgical complication during in-hospital stay were also assessed. The patients were followed for 6 months and evaluated using the Glasgow Outcome Scale (GOS). The follow-up consultations were carried out at the HC-FMUSP Vascular Neurosurgery Clinic or by phone calls.

### Statistical analysis

Descriptive statistics were applied to describe the clinical and demographic characteristics of the patients studied. Continuous variables were described as mean (standard deviation) or median (interquartile distance), as appropriate under normal data analysis. Categorical data were described as frequencies (valid percentage). Logistic regression was used to assess predictors of poor outcomes in short-term outcomes (clinical and surgical complications). Predictors of long-term outcomes (GOS) were assessed through ordered logistic regression. A two-tailed alpha level of 5% was adopted. The analyses were performed using the Stata software, version 17.0. Data from microsurgical and endovascular group were analyzed separately and the results were subsequently compared and discussed.

## Results

**Figure 1:**
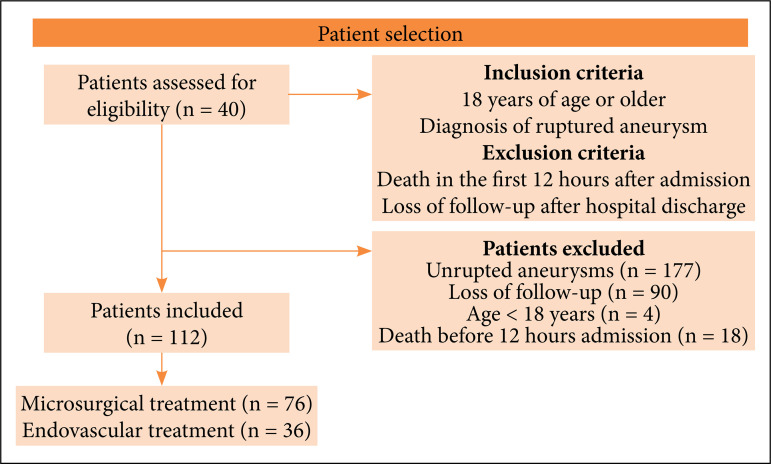
Flowchart of patient selection.

A total of 112 patients were included. Seventy-six were submitted to microsurgical treatment and thirty-six to endovascular treatment (Fig. 1). The overall comparison between groups demonstrated no significant difference in terms of death at discharge (p = 0.06) and GOS in the follow-up (p = 0.44).

### Microsurgical group

The microsurgical group had a mean age (SD) of 55.69 ± 12.43 and included 54 (71%) females, 22 (28.9%) males, 39 (51.3%) patients with hypertension, 18 (23.6%) with diabetes mellitus, 7 (9.2%) with a previous SAH, 9 (11.8%) with alcohol abuse and 30 (39.4%) were smokers (Tables 1 and 2). The median (interquartile range, IQR) period between ictus and surgery was 5 ± 9 days. Fifty-three (69.7%) patients had HH ≤ 2 on admission and 23 (30.2%) HH > 2 on admission.

**Table 1:** Demographic characteristics and its respective association with GOS score in follow-up.

	Microsurgical group	p-value	Endovascular group	p-value
Total n (%)	76 (100.0%)	–	36 (100.0%)	–
Age mean (SD)	55.7 (12.4)	0.32	58.4 (14.3)	0.01
Female n (%)	54 (71.0%)	0.72	25 (69.4%)	0.77
Hypertension n (%)	39 (51.3%)	0.03	19 (52.7%)	0.001
Diabetes mellitus n (%)	18 (23.6%)	0.7	10 (27.7%)	0.07
Previous SAH n (%)	7 (9.2%)	0.57	5 (13.8%)	0.98
Smoking n (%)	30 (39.4%)	0.81	9 (25.0%)	0.80
Alcohol abuse n (%)	9 (11.8%)	0.96	7 (19.4%)	0.29

**Table 2:** Demographic characteristics and its respective association with death at discharge.

	Microsurgical group	p-value	Endovascular group	p-value
Total n (%)	76 (100.0%)	–	36 (100.0%)	–
Age mean (SD)	55.7 (12.4)	0.87	58.4 (14.3)	0.03
Female n (%)	54 (71.0%)	0.03	25 (69.4%)	0.75
Hypertension n (%)	39 (51.3%)	0.05	19 (52.7%)	0.36
Diabetes mellitus n (%)	18 (23.6%)	0.08	10 (27.7%)	0.99
Previous SAH n (%)	7 (9.2%)	0.88	5 (13.8%)	0.99
Smoking n (%)	30 (39.4%)	0.66	9 (25.0%)	0.39
Alcohol abuse n (%)	9 (11.8%)	0.97	7 (19.4%)	NA

NA: not assessed. ^a^No patient who underwent endovascular treatment and died at discharge had history of alcohol abuse.

The median (IQR) size (i.e., the largest dimension) of the aneurysms was 6±5 mm, 19 (25%) patients had atherosclerotic plaque, 12 (15.7%) patients had multiple aneurysms. MFisher was assessed on 75 patients: 20 (26.6%) had mFisher 0–2, 26 (34.6%) had mFisher 3, 29 (38.6%) had mFisher 4. Regarding the ruptured aneurysm location: n = 27 (35.5%) were in PCoA, n = 24 (31.5%) were in MCA, n = 12 (15.7%) were in anterior communication artery (ACoA), n = 5 (6.5%) were in ICA and n = 8 (10.5%) were in other arteries, which included ACA, PICA, pericallosal, AICA, ophthalmic, vertebral and basilar arteries (Tables 3 and 4).

**Table 3:** Radiological features and GOS in follow-up.

	Microsurgical group	p-value	Endovascular group	p-value
Total n (%)	76 (100.0%)	–	36 (100.0%)	–
**Modified Fisher^a^**				
mFisher 0 n (%)	3 (3.9%)	NA	1 (2.0%)	NA
mFisher 1 n (%)	7 (9.2%)	0.66	6 (16.0%)	NA
mFisher 2 n (%)	3 (3.9%)	0.57	0 (0.0%)	NA
mFisher 3 n (%)	26 (34.6%)	0.02	9 (26.4%)	0.03
mFisher 4 n (%)	29 (38.6%)	0.15	18 (52.9%)	0.56
**Aneurysm location**				
PCoA n (%)	27 (35.5%)	0.19	5 (13.8%)	NA
MCA n (%)	24 (31.5%)	0.14	3 (8.3%)	NA
ACoA n (%)	12 (15.7%)	0.98	8 (22.2%)	0.46
ICA n (%)	5 (6.5%)	0.74	9 (25.0%)	0.12
Others n (%)	8 (10.5%)	0.92	11 (30.5%)	0.95
**Aneurysm size median (IQR)**	6 ± 5	0.05	5 ± 5	0.06
Multiple n (%)	12 (15.7%)	0.03	6 (16.6%)	0.78
Plaque n (%)	19 (25.0%)	0.03	12 (33.3%)	0.38

a mFisher was assessed in 75 patients from the microsurgical group and 34 patients from the endovascular group. NA: no association demonstrated statistically due to nonachievement of statistical convergence.

**Table 4:** Radiological features and death at discharge. mFisher was assessed in 75 patientsfrom the microsurgical group and 34 patients from the endovascular group.

	Microsurgical group	p-value	Endovascular group	p-value
Total n (%)	76 (100.0%)	–	36 (100.0%)	–
**Modified Fisher^a^**				
mFisher 0 n (%)	3 (3.9%)	NA	1 (2.0%)	NA
mFisher 1 n (%)	7 (9.2%)	0.85	6 (16.0%)	NA
mFisher 2 n (%)	3 (3.9%)	0.63	0 (0.0%)	NA
mFisher 3 n (%)	26 (34.6%)	0.14	9 (26.4%)	NA
mFisher 4 n (%)	29 (38.6%)	NA	18 (52.9%)	NA
**Aneurysm location**				
PCoA n (%)	27 (35.5%)	0.36	5 (13.8%)	NA
MCA n (%)	24 (31.5%)	0.11	3 (8.3%)	NA
ACoA n (%)	12 (15.7%)	0.01	8 (22.2%)	NA
ICA n (%)	5 (6.5%)	NA	9 (25.0%)	NA
Others n (%)	8 (10.5%)	NA	11 (30.5%)	NA
**Aneurysm size median (IQR)**	6 ± 5	0.14	5±5	0.98
Multiple n (%)	12 (15.7%)	0.66	6 (16.6%)	NA
Plaque n (%)	19 (25.0%)	0.02	12 (33.3%)	NA

NA: no association demonstrated statistically due to nonachievement of statistical convergence.

a mFisher was assessed in 75 patients from the microsurgical group and 34 patients from the endovascular group.

Eighteen (23.6%) patients were dead at discharge. Among the 58 patients followed 6 months later, 4 (6.8%) died after discharge (GOS 1), 2 (3.4%) presented severe disability (GOS 3), 14 (24.1%) had minor neurologic deficits (GOS 4) and 38 (65.5%) returned to their original functional level (GOS 5) (Table 5). The statistical analysis revealed that hypertension (p = 0.03; 95% confidence interval [CI] = 0.12–2.62), mFisher 3 (p = 0.02; 95% CI = 0.33–6.19), presence of atherosclerotic plaque (p = 0.03; 95% CI = 0.09–2.80) and multiple aneurysms (p = 0.03; 95% CI = 0.10–4.05) were associated with GOS in the follow-up. Female gender (odds ratio [OR] = 0.25; p = 0.03; 95% CI = 0.072–0.92), ACoA location (OR = 14935.17; p = 0.01; 95% CI = 4.86–4.58) and presence of atherosclerotic plaque (OR = 179.22; p = 0.02; 95% CI = 2.03–15789.2) were associated with death at discharge.

**Table 5:** Short and long-term outcomes.

	Microsurgical group	Endovascular group
Total n (%)	76 (100.0%)	36 (100.0%)
**Death at discharge n (%)**	18 (23.6%)	11 (30.5%)
**GOS in follow-up**		
GOS 1 n (%)	4 (5.2%)	4 (11.1%)
GOS 2 n (%)	0 (0.0%)	0 (0.0%)
GOS 3 n (%)	2 (2.6%)	5 (13.8%)
GOS 4 n (%)	14 (18.4%)	4 (11.1%)
GOS 5 n (%)	38 (50.0%)	12 (33.3%)

Endovascular group

The endovascular group had a mean age (SD) of 58.39 (14.3) and included 25 (69.4%) females, 11 males (30.5%), 19 (52.7%) patients with hypertension, 10 (27.7%) with diabetes mellitus, 5 (13.8%) with a previous SAH, 7 (19.4%) with alcohol abuse and 9 (25%) were smokers (Tables 1 and 2). The median (IQR) period between ictus and surgery was 5 ± 5 days. Twenty-two (61.1%) patients had HH ≤ 2 on admission and 14 (38.8%) HH > 2 on admission.

The median (IQR) size (i.e., the largest dimension) of the aneurysms was 5±5 mm, n = 12 (33.3%) patients had atherosclerotic plaque, n = 6 (16.6%) patients had multiple aneurysms. MFisher was assessed on 34 patients: n = 7 (20.5%) had mFisher 0–2, n = 9 (26.4%) mFisher 3, n = 18 (52.9%) mFisher 4. Regarding the ruptured aneurysm location: n = 5 (13.8%) were in PCoA, n = 3 (8.3%) were in MCA, n = 8 (22.2%) were in ACoA, n = 9 (25%) were in ICA and n = 11 (30.5%) were in other arteries, which included ACA, PICA, Pericallosal, AICA, Ophthalmic, vertebral and basilar arteries (Tables 3 and 4).

Eleven (30.5%) patients were dead at discharge. Among the remaining 25 patients who were followed 6 months later, 4 (16%) had died during the follow-up period (GOS 1), 5 (20%) presented severe disability (GOS 3), 4 (16%) had minor neurologic deficits (GOS 4) and 12 (48%) had returned to their original functional level (GOS 5) (Table 5).

The statistical analysis revealed that age (p = 0.01; 95% CI = 0.23–0.02) hypertension (p = 0.001; 95% CI = 2.09–8.02) and mFisher 3 (p = 0.03; 95% CI = 11.12–0.52) were associated with clinical outcome in follow-up. Age (OR = 1.11; p = 0.039; 95% CI = 1.00–1.23) was also associated with death at discharge.

## Discussion

In this study, death at discharge was associated with female gender, ACoA aneurysm location and presence of atherosclerotic plaque in patients surgically treated, while only age was associated with the same outcome in patients who underwent the endovascular procedure. Both groups had clinical condition on 6 months of follow-up associated with mFisher score on admission and hypertension. Additionally, the multivariate analysis demonstrated that the presence of atherosclerotic plaque on imaging and multiple aneurysms could predict clinical long-term outcomes in patients treated surgically, while only age was associated with long-term outcomes in the endovascular group.

Studies have shown that higher mFisher grade and hypertension are associated with poor long-term outcomes in patients with aSAH, regardless of the type of treatment^7^–^11^. The same pattern was observed in this study. Premorbid hypertension is an independent risk factor for increased severity of aSAH, rebleeding, hydrocephalus, mortality, and disability^7^–^13^. The association between poor prognosis and female gender is likely due to the higher risk of vasospasm after aSAH in females^14^.

The association between systemic hypertension and intracranial aneurysm formation and ruptured is well established, but the mechanism behind this association is not fully understood. The link between systemic hypertension and IA might be the renin-angiotensin system (RAS). RAS plays an important role in vasorelaxation/vasoconstriction, vascular remodeling, and maintenance of arterial wall integrity^15^–^17^. Zheng *et al*.^12^ retrospectively studied hypertensive patients with aSAH and showed that those treated with angiotensin-converting enzyme inhibitors and angiotensin receptor blockers suffered from less amount of aneurysmal bleeding than the patients treated with other types of antihypertensive drugs.

Although the neurological status on admission is considered the most important predictor for aSAH outcome, this study found no association between initial HH scores and short- or long-term outcomes for both groups^10^,^18^,^19^. On the other hand, age seemed to be the main predictor of poor outcomes in patients who underwent embolization. In fact, older age has been previously associated with increased mortality and discharge to long-term facilities in patients with SAH^20^–^23^. Proust *et al.* 24 assessed clinical outcomes of elderly patients (70 years or older) with ruptured intracranial aneurysms after the surgical or endovascular treatment, concluding that age exceeding 75 years was associated with functional dependence for groups.

The aneurysm location and size are also important factors when choosing between the two procedures. Some studies indicate that PCoA and MCA location are risk factors for intraprocedural rupture and poor outcomes when coiled^5^,^25^. On the other hand, endovascular approach of lesions < 3 mm is more prone to evolve to intraprocedural rupture^6^. In this study, the aneurysm size and location were not associated with outcomes in the endovascular group, which most likely reflect the limited sample size than the absence of association *per se*. Additionally, most aneurysms were bigger than 3 mm, limiting the analysis. However, the surgical group had a positive association between death at discharge and ACoA aneurysm location.

The presence of atherosclerotic plaque on imaging was associated to poor clinical outcomes 6-months later in the group treated surgically. The pathophysiology behind the unfavorable outcomes of patients treated with aneurysms treated via surgery when compared with those treated via embolization was not yet clarified or even sufficiently investigated. The finding of this study suggest that the surgical treatment may lead to the inadvertent mobilization of intravascular atherosclerotic plaques, leading to microembolization and microinfarcts.

Multiple aneurysms were also predictors of poor outcomes in follow-up. Few studies have evaluated the impact of multiple lesions on long-term outcomes, most of them suggest that patients with unruptured multiple lesions do not have increased risk of rupture or poor clinical outcomes^26^, ^27^. However, as this study investigated a subset of subjects who already suffered the rupture, it suggests that, once evolving with aSAH, cases of multiple aneurysms have higher risk of unfavorable outcomes.

## Limitations

This study presents limitations that should be considered aiming a proper interpretation. First, the results’ analysis is limited by the small sample size, especially in the endovascular group. Second, the loss of follow-up, due to impossibility of following long-distance patients and giving up the participation in the study, limited the long-term analysis. Finally, the inconsistent results regarding the association of aneurysm size and location with clinical outcomes may be explained by the treatment selection bias.

## Conclusions

Premorbid hypertension and severity of aSAH on imaging are predictors of poor outcomes, regardless of the type o treatment undergone. Subjects treated surgically are more susceptible to unfavorable outcomes if atherosclerotic plaques are present on imaging. This finding suggests that the vessel manipulation during the surgical procedure may cause inadvertent mobilization of atherosclerotic lesions, leading to detachment of microemboli into the cerebral circulation. Studies about pathophysiology of this process, which may explain the better results of endovascular procedures in many cases, are still lacking. Multiple aneurysms in the context of aSAH were associated with poor outcomes when treated surgically. In patients who underwent endovascular treatment, age was the main predictor of clinical outcome.
